# Corticospinal Modulations during Bimanual Movement with Different Relative Phases

**DOI:** 10.3389/fnhum.2016.00095

**Published:** 2016-03-07

**Authors:** Yoshifumi Nomura, Yasutomo Jono, Keisuke Tani, Yuta Chujo, Koichi Hiraoka

**Affiliations:** ^1^Graduate School of Comprehensive Rehabilitation, Osaka Prefecture UniversityHabikino, Japan; ^2^College of Health and Human Sciences, Osaka Prefecture UniversityHabikino, Japan

**Keywords:** bimanual coordination, relative phase, attractor state, intrinsic dynamics, corticospinal excitability

## Abstract

The purpose of this study was to investigate corticospinal modulation of bimanual (BM) movement with different relative phases (RPs). The participants rhythmically abducted and adducted the right index finger (unimanual (UM) movement) or both index fingers (BM movement) with a cyclic duration of 1 s. The RP of BM movement, defined as the time difference between one hand movement and the other hand movement, was 0°, 90°, or 180°. Motor evoked potentials (MEPs) in the right flexor dorsal interosseous muscle elicited by transcranial magnetic stimulation (TMS) were obtained during UM or BM movement. Corticospinal excitability in the first dorsal interosseous muscle during BM movement with 90° RP was higher than that during UM movement or BM movement with 0° or 180° RP. The correlation between muscle activity level and corticospinal excitability during BM movement with 90° RP was smaller than that during UM movement or BM movement with 0° or 180° RP. The higher corticospinal excitability during BM movement with 90° RP may be caused by the greater effort expended to execute a difficult task, the involvement of interhemispheric interaction, a motor binding process, or task acquisition. The lower dependency of corticospinal excitability on the muscle activity level during BM movement with 90° RP may reflect the minor corticospinal contribution to BM movement with an RP that is not in the attractor state.

## Introduction

Bimanual (BM) movement can be performed with various time differences between one hand movement and the other hand movement. This time difference is referred to as the relative phase (RP), and is expressed as the phase angle relative to a 360° movement cycle; i.e., the RP of the in-phase BM movement is 0° and that of the anti-phase BM movement is 180°. It has been well established that BM movement with 0° or 180° RP is stable and accurate (Zanone and Kelso, 1992). In contrast, the error and variability of the observed RP are relatively large during BM movement with RPs other than 0° or 180° (Yamanishi et al., 1980; Tuller and Kelso, 1989; Zanone and Kelso, 1992; Lee et al., 1995; James et al., 2010). Moreover, the observed RP during BM movement with a required RPs other than 0° and 180° gets closer to the 0° or 180° RP (Yamanishi et al., 1980; Zanone and Kelso, 1992; Lee et al., 1995; James et al., 2010). These findings are explained by the locus of the attractors according to dynamic pattern theory (Haken et al., 1985); attractors are intrinsic dynamics that organize movement patterns in stable and accurate attractor states that are located in the 0° and 180° RPs (Zanone and Kelso, 1992). Based on this theory, the greater error and variability observed during BM movement with an RP other than 0° or 180° can be explained by the fact that the RP is not in the attractor state.

Neural interactions between the limbs occur when the two limbs move simultaneously (Zehr and Duysens, 2004; Carson, 2005). According to a study using functional MRI (fMRI) and transcranial magnetic stimulation (TMS), the right superior temporal gyrus plays a role in BM coordination (Duque et al., 2010). Neural interaction between the control of one limb and that of the other limb is dependent on the RP (Haken et al., 1985; Court et al., 2002). Indeed, the corticospinal excitability of the flexor carpi radialis (FCR) muscle was differently modulated during isodirectional and anisodirectional movements of the wrist and ankle (McIntyre-Robinson and Byblow, 2013). The posterior parietal cortex and the pre-supplementary motor area are activated during asymmetrical BM movement in healthy humans or in patients with right-brain-damage without motor neglect (Garbarini et al., 2014, 2015). Moreover, each of the supplementary motor area, premotor area, inferior frontal gyrus, post-central gyrus, basal ganglia, and cerebellum play roles in controlling BM movement with 180° RP (Sadato et al., 1997; Steyvers et al., 2003; Wu et al., 2010; Wilson et al., 2014). In spite of these previous findings, the neural interaction during BM movement with RPs that are not in the attractor state is not well understood.

Interhemispheric interaction contributes to BM movement with the RPs that are not in the attractor state, according to a previous finding that BM movement with RPs other than 0° and 180° is less stable in split-brain patients than healthy humans (Tuller and Kelso, 1989). The primary motor cortices (M1s) are interconnected by the corpus callosum mediating interhemispheric interaction (Rouiller et al., 1994; Wahl et al., 2007); e.g., interhemispheric inhibition of the M1 is induced by BM coordination of force (Hiraoka et al., 2014), and most neurons in the M1 show activity specific to BM movements (Donchin et al., 1998). Thus, the M1s must be involved in the process of controlling BM coordination (Donchin et al., 1998). The contributions of the M1 to BM movement are dependent on the RP. A previous study using fMRI showed that the activity of the right M1 during BM movement with 180° RP is less than that during BM movement with 0° RP (Aramaki et al., 2006). The interference of anti-phase BM tapping caused by TMS over the M1 is smaller than that of in-phase BM tapping (Chen et al., 2005). Therefore, modulation of the corticospinal pathway, which involves the M1, during BM movement may be dependent on the RP.

Supraspinal control of BM movement with the RPs in the attractor state has been investigated using TMS that elicits motor evoked potential (MEP). MEP in the FCR muscle during BM movement with 180° RP was larger than that during BM movement with 0° RP in three out of four participants, although this difference was canceled when the MEP amplitude was normalized with respect to the electromyographic (EMG) level (Carson et al., 1999). In spite of this previous finding, corticospinal modulation during BM movement with an RP which is not in the attractor state has not been investigated. Corticospinal excitability in the FDI muscle during a precision task was higher when force adjustment was difficult (Pearce and Kidgell, 2009). Asynergistic contractions of the bilateral forearm muscles reduced corticospinal excitability, but this inhibitory process was inactivated during mirror contractions of those muscles, indicating that corticospinal excitability during difficult bilateral movement is different from that during relatively easy bilateral movement (Leonard et al., 2015). Accordingly, corticospinal excitability during BM movement with an RP which is not in the attractor state may be different from that during BM movement with an RP in the attractor state, because of task difficulty.

Corticospinal excitability of the forearm muscle was shown to be phase-dependently facilitated by rhythmic movement of the contralateral wrist (Carson et al., 2004). This facilitation was prominent in the phase in which the contralateral homogeneous muscle was active, indicating that corticospinal excitability in the tested muscle increases with activation of the contralateral homologous muscle. The phase of the tested limb movement, in which the muscle contralateral to the tested limb is active, shifts in accordance with the RP during BM movement. Given this fact, it was hypothesized that the phase in which the corticospinal excitability increases during BM movement would differ according to the RP. That is, corticospinal excitability would be increased in the abduction phase of finger movement during BM movement with 0° RP, would be relatively high in the adduction phase of finger movement during BM movement with 180° RP, and would be relatively high in the transition phase from abduction to adduction of finger movement during BM movement with 90° RP. In the present study, therefore, we investigated the modulation of corticospinal excitability during BM movement with different RPs.

## Materials and Methods

### Participants

The participants were 10 healthy males aged 22–41 years (30.6 ± 6.6 years). Only males were recruited to rule out across-participants variability of BM coordination caused by unknown sex-related influences. All participants were right-handed according to the Edinburgh Handedness Inventory (Oldfield, 1971). All of the participants had no history of neurological or orthopedic diseases. All of the experimental procedures were approved by the ethics committee of Osaka Prefecture University.

### Apparatus

The participants were seated in front of a table. A monitor, indicating a lissajous figure of a target point trajectory and that of a trajectory of actual finger movement, was placed 1 m in front of the participants. The forearms were pronated with the palms faced downward. The hands were placed over the devices preventing movement of the fingers other than the index fingers. Abduction-adduction movements of the index fingers were measured by electrogoniometers placed over the index fingers. The signals from the electrogoniometers were amplified with strain amplifiers (PH-412B; DKH, Tokyo, Japan). Ag/AgCl surface electrodes recording EMG signals were placed over the FDI muscles using a belly-tendon montage. The EMG signals were amplified by an amplifier (MEG-2100; Nihon Kohden, Tokyo, Japan) with passband filters of 15 Hz–3 kHz. The signals were converted to digital signals using A/D converters (PowerLab 800S: AD Instruments, Colorado Springs, USA; Unique Acquisition UAS-A1: Unique Medical, Tokyo, Japan) at a sampling rate of 10 kHz and stored in personal computers.

### TMS

Monophasic TMS was delivered using a figure-of-eight coil with an outer diameter of 99 mm (YM-133B; Nihon Kohden) connected to a magnetic stimulator (SMN-1200; Nihon Kohden). The maximum intensity of the coil was 0.96 T. The coil was placed tangentially to the scalp at a 45° angle to the sagittal plane inducing posterior-anterior electrical current in the brain. The hotspot of the right FDI muscle was determined by searching the site where the maximum MEP amplitude was obtained. The resting motor threshold of the right FDI muscle was the minimal intensity of TMS producing an MEP amplitude larger than 50 μV in 5 out of 10 stimulations delivered over the hotspot. The TMS intensity was 10% above the intensity of TMS at the resting motor threshold.

### Procedure

**Figure 1:**
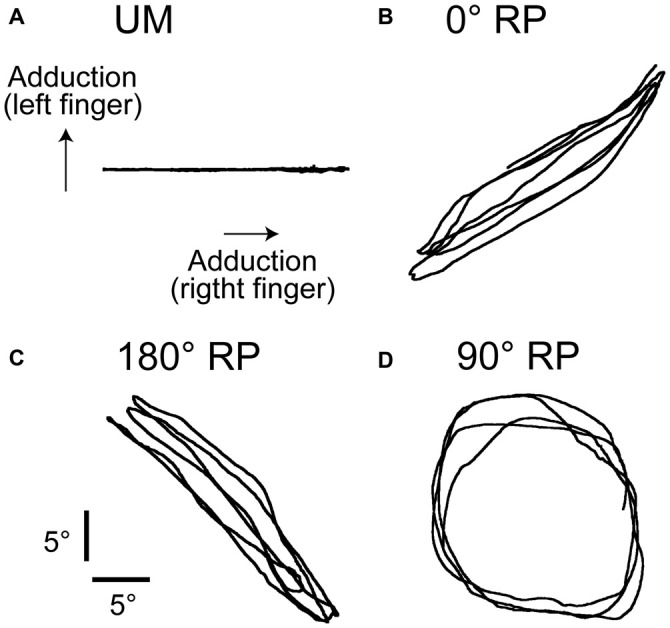
**Lissajous figures of the actual movement trajectory during unimanual (UM) movement (A) or bimanual (BM) movement with 0° (B), 180° (C), or 90° of the relative phase (RP; D) in the non-transcranial magnetic stimulation (non-TMS) session.** The traces indicate the angle of the index finger.

The participants rhythmically abducted and adducted the right index finger or both index fingers with the cyclic duration of 1 s. The participants traced a visual target line (2 mm thickness) with a circle-shaped cursor (5 mm diameter). The displacement of the cursor to the right indicated adduction of the right index finger and that to the top indicated adduction of the left index finger (Figure 1). The lissajous figure of the cursor indicating actual finger movements has been shown to help participants to execute BM movement with 90° RP (Verschueren et al., 1997; Kovacs et al., 2009; Kovacs and Shea, 2011). The participants traced a horizontal target line with 18 cm length to execute unimanual (UM) movement with the right index finger (UM condition) as shown in Figure 1A, traced a right up diagonal linear target line with 25.4 cm length to execute BM movement with 0° RP (0° RP condition) as shown in Figure 1B, traced a right down diagonal linear line with 25.4 cm length to execute BM movement with 180° RP (180° RP condition) as shown in Figure 1C, and traced a circle target line with 16 cm diameter in the clockwise direction to execute BM movement with 90° RP (90° RP condition) as shown in Figure 1D. The peak angle of each finger during the task, indicating the extreme of each target line, was adjusted to an appropriate number of degrees so that the participants could comfortably repeat the finger movement without fatigue during the experimental sessions. The auditory high tone (1 kHz) and the auditory low tone (166 Hz) were alternately generated with a 500 ms interval so that the participants could take movement timing (Kelso and Schöner, 1988; Lee et al., 1995; Carson et al., 2004). The participants adducted the right finger when the auditory high tone was given and abducted the right finger when the auditory low tone was given.

The TMS session was conducted after the non-TMS session. In the non-TMS session, background EMG (BEMG) and finger movement were recorded for 100 cycles of successful finger movement in each task. The order of tasks to be performed was randomly assigned each participant in the non-TMS session. In the TMS session, TMS was delivered every 5300 ms until 100 of MEPs were recorded for each task. The tasks were randomly ordered for each session for each participant in the TMS session. The trials in which the rhythm of finger movement was completely outside the movement cycle paced by the auditory tones were considered to be error trials and were excluded from the successful trials online. In the TMS session, the finger movement cycle was divided into four phases of adduction of the index finger (from the 1st to 4th phase) and four phases of abduction of the index finger (from the 5th to 8th phase) offline, as previous studies divided the wrist movement cycle into eight phases (Carson et al., 1999, 2004). The 1st phase was the beginning of adduction of the index finger and the 8th phase was the end of abduction of the index finger. The duration of the movement phase was 125 ms each. Thus, theoretically, 12–13 trials could be obtained in each phase of each task in the TMS session. BM movement with 90° RP is difficult, but improves with practice (Zanone and Kelso, 1992; Lee et al., 1995). Therefore, before beginning the sessions, the participants practiced the tasks until they could perform them properly. An experimenter monitored the display, assessed the performance of movement online, and determined when to finish the practice.

### Data Analysis in Non-TMS Session

**Figure 2:**
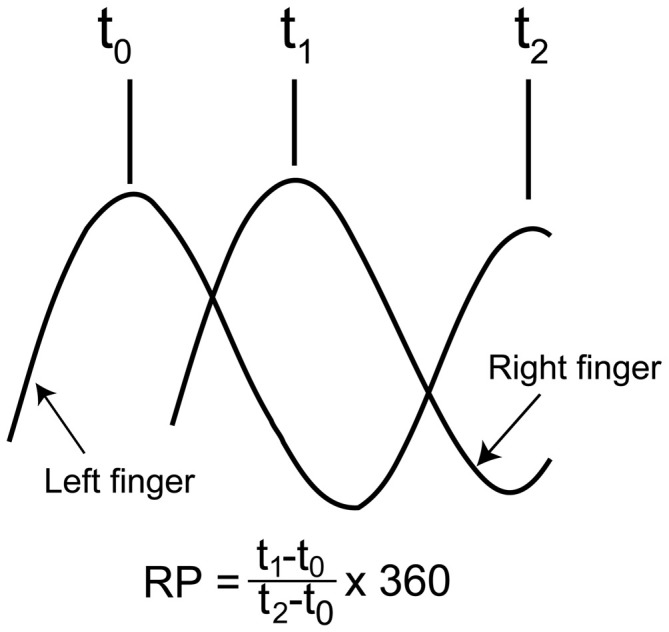
**Schematic explanation for calculation of the relative phase (RP).** As shown in the expression, the time of the peak abduction of the right finger is subtracted from that of the left finger, and this is expressed as degrees relative to the degree of the right finger movement cycle.

The time difference between the occurrence of peak abduction of the right finger and that of the left finger closest in time was calculated first, and this time difference was expressed as the degrees relative to the period of the right finger cycle (RP), as shown in Figure 2 (Zanone and Kelso, 1992; James et al., 2010). The absolute difference between the required RP and the observed RP (absolute delta RP) and the SD of the absolute delta RP were estimated in the non-TMS session (Verschueren et al., 1997; Kovacs et al., 2009; Kovacs and Shea, 2011). The absolute delta RP indicates the accuracy of bilateral movement, because it represents the deviation of the observed RP from the required RP (Salter et al., 2004; Kovacs et al., 2009). The SD of the absolute delta RP indicates the stability of bilateral movement, because this represents the variability in the deviation of the observed RP from the required RP (Kovacs et al., 2009). A total of 100 trials in the non-TMS session were divided into five trial blocks, i.e., trials number 1–20, 21–40, 41–60, 61–80, and 81–100, and the statistical significance of changes in the absolute delta RP and SD of the absolute delta RP were examined across the trial blocks. This analysis was made to elucidate whether acquisition of the task occurred during the non-TMS session. The EMG was rectified and then averaged for each movement cycle starting at the onset of adduction of the right finger movement and ending at the offset of abduction of the right finger movement.

### Data Analysis in TMS Session

The MEP amplitude was estimated on a peak-to-peak basis. The pre-stimulus BEMG amplitude was expressed as the average of the route mean square of the EMG amplitude in the time window from 110 to 10 ms before TMS. The angle and angular velocity of the metacarpophalangeal joint of the index finger were also estimated in the time window from 110 to 10 ms before TMS.

### Statistics

Measurements for the four tasks, the UM movement and the BM movement with 0°, 180°, and 90° RPs, were statistically analyzed in the right finger movement or BEMG in the right FDI muscle. On the other hand, statistical analysis for the left finger movement or BEMG for the left FDI muscle were performed on the three BM tasks with 0°, 180° and 90° RPs, since the left finger was at rest during the UM condition. Pearson’s correlation coefficient between the pre-stimulus BEMG amplitude and MEP amplitude was estimated for each movement phase and across the movement phases. One-way repeated measures analysis of variance (ANOVA) was conducted to test the effect of the task on the correlation coefficient between the pre-stimulus BEMG and MEP amplitudes across the movement phases [4 (task)]. In addition, one-way ANOVA was conducted to test the effect of the task on the number of the error trials in the TMS session [4 (task)]. Similarly, one-way ANOVA was conducted to test the effect of task on the right finger movement amplitude [4 (task)], and another one-way ANOVA was conducted to test the effect of the task on the amplitude of left finger movement [3 (task)]. Two-way repeated measures ANOVA was conducted to test the effect of the movement phase and that of the task on the angle or velocity of the left index finger movement and on the pre-stimulus BEMG amplitude in the left FDI muscle [8 (movement phase) × 3 (task)], Similarly, two-way ANOVA was conducted to test the effect of the trial block and task on the absolute delta RP and SD of absolute delta RP [5 (trial block) × 3 (task)]. In addition, two-way ANOVA was conducted to test the effect of the movement phase and task on the angle or velocity of right finger movement [8 (movement phase) × 4 (task)]. Another two-way ANOVA was conducted to test the effect of the movement phase and task on the pre-stimulus BEMG amplitude, MEP amplitude, or the correlation coefficient between the pre-stimulus BEMG and MEP amplitudes for each movement phase in the right FDI muscle [8 (movement phase) × 4 (task)]. When ANOVA revealed a significant interaction between the main effects, a test of the simple main effect was conducted. When ANOVA or the test of the simple main effect revealed a statistically significant difference, a multiple comparison test (Bonferroni test) was conducted. The alpha level was 0.05 for these analyses. All data were expressed as means and standard errors of the mean.

## Results

**Figure 3:**
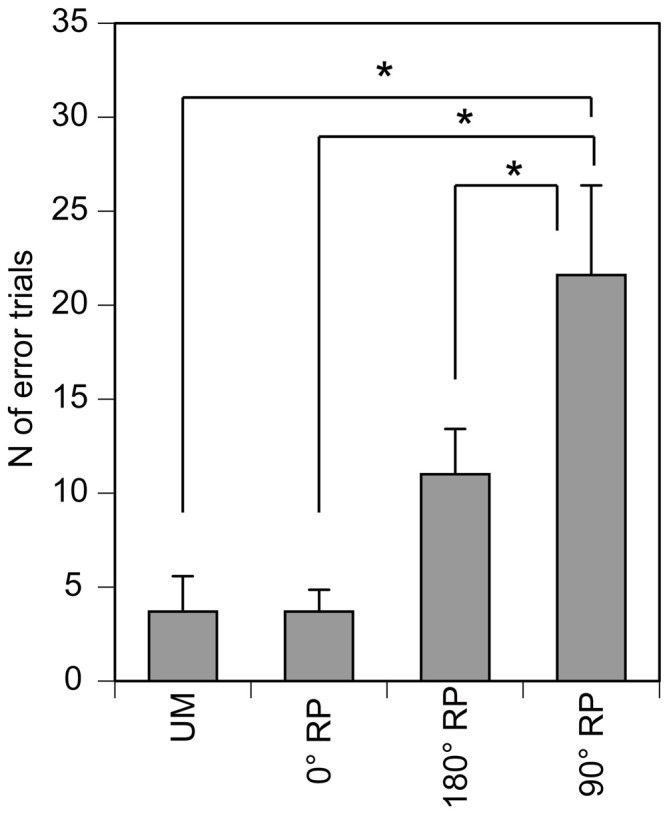
**The number of the error trials in the TMS session.** Bars indicate means and error bars indicate standard errors. Asterisks indicate significant differences (*p* < 0.05).

All participants could perform any of the four tasks after practice. The number of successful trials in each phase for each task was 12.5 ± 0.2 in the TMS session. The number of the error trials was 3.7 ± 1.9 under the UM condition, 3.7 ± 1.1 under the 0° RP condition, 11.0 ± 2.4 under the 180° RP condition, and 21.6 ± 4.8 under the 90° RP condition (Figure 3). ANOVA revealed a significant difference in the number of the error trials across the tasks [*F*_(3,27)_ = 11.72, *p* < 0.01]. The *post hoc* test revealed that the number of error trials under the 90° RP condition was significantly larger than that under any of the other tasks (*p* < 0.05).

### Finger Movement Angle

**Figure 4:**
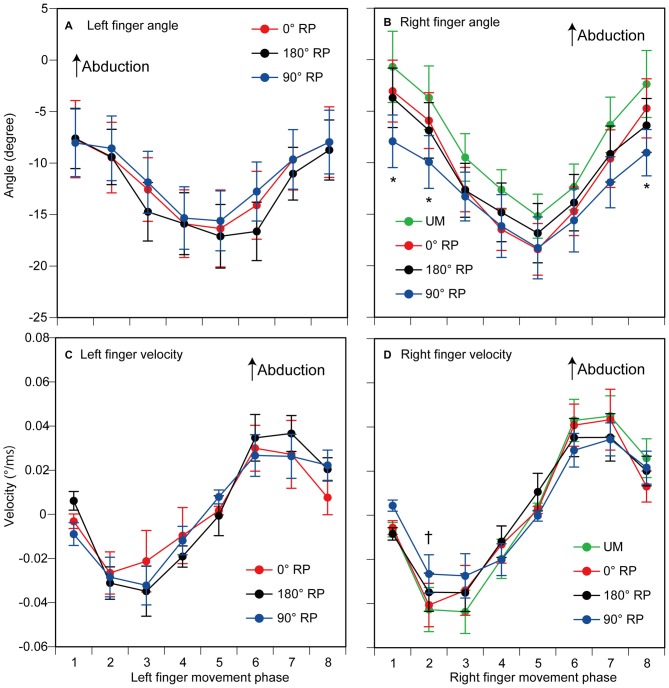
**The angle (A,B) and the velocity of index finger movement (C,D) in the TMS session.** The left panels are data from the left finger **(A,C)** and the right panels are data from the right finger **(B,D)**. Data points indicate means and error bars indicate standard errors. Asterisks and a dagger indicate the movement phases where significant difference is found among the tasks (*p* < 0.05). Please see the text for details.

The peak abduction of the finger appeared in the 1st and 2nd phases and the peak adduction of the finger appeared in the 5th phase in the TMS session (Figures 4A,B). ANOVA revealed a significant difference in the angle of the left index finger among the phases [*F*_(7,63)_ = 10.45, *p* < 0.01] but did not reveal such a difference among the tasks [*F*_(2,18)_ = 1.48, *p* = 0.26] without a significant interaction between the main effects [*F*_(14,126)_ = 0.76, *p* = 0.71] (Figure 4A). ANOVA revealed a significant difference in the angle of the right index finger among the phases [*F*_(7,63)_ = 29.13, *p* < 0.01], but failed to reveal a significant difference among the tasks [*F*_(3,27)_ = 2.11, *p* = 0.12] with a significant interaction between the main effects [*F*_(21,189)_ = 2.28, *p* < 0.01] (Figure 4B). The test of the simple main effect revealed a significant difference among the tasks in the 1st [*F*_(3,38)_ = 3.97, *p* = 0.01] and 2nd phases [*F*_(3,38)_ = 2.93, *p* < 0.05]. The *post hoc* test revealed that the angle was significant different among any pair of the tasks except between BM movement with 0° RP and that with 180° RP both in the 1st and 2nd phases.

### Finger Movement Velocity

The velocity of each finger movement in each phase is shown in Figures 4C,D. ANOVA revealed a significant difference in the velocity of the left finger movement among the phases [*F*_(7,63)_ = 10.37, *p* < 0.01], but did not reveal such a difference among the tasks [*F*_(2,18)_ = 0.23, *p* = 0.80] without a significant interaction between the main effects [*F*_(14,126)_ = 0.82, *p* = 0.65] (Figure 4C). ANOVA revealed a significant difference in the velocity of the right finger movement among the phases [*F*_(7,63)_ = 15.05, *p* < 0.01], but did not reveal a significant difference among the tasks [*F*_(3,27)_ = 0.13, *p* = 0.94] with a significant interaction between the main effects [*F*_(21,189)_ = 2.06, *p* < 0.01] (Figure 4D). The test of the simple main effect revealed a significant difference among the tasks in the 2nd phase [*F*_(3,214)_ = 2.79, *p* = 0.04]. A *post hoc* test revealed that the velocity was significantly different among any pair of the tasks except between the UM movement and BM movement with 0° RP or that with 180° RP.

### Finger Movement Amplitude

**Figure 5:**
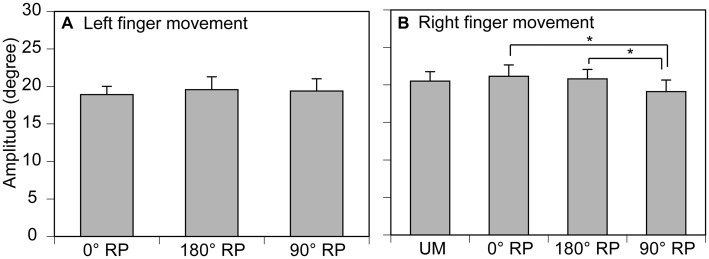
**Movement amplitude in the left (A) and right fingers (B) in the non-TMS session.** Bars indicate means and error bars indicate standard errors. Asterisks indicate significant differences (*p* < 0.05).

The amplitude of finger movement was 20.4 ± 0.7 degrees in the left finger (moving) and 19.3 ± 0.8 degrees in the right finger in the non-TMS session (Figure 5). The amplitude of left finger movement (moving) was not significantly different among the tasks [*F*_(2,18)_ = 0.41, *p* = 0.67]. In contrast, the amplitude of right finger movement was significantly different among the tasks [*F*_(3,27)_ = 4.58, *p* = 0.01]. A *post hoc* test revealed that the amplitude during BM movement with 90° RP was significantly smaller than that during BM movement with 0° RP or that during BM movement with 180° RP (*p* < 0.05).

### Accuracy of Finger Movement

**Figure 6:**
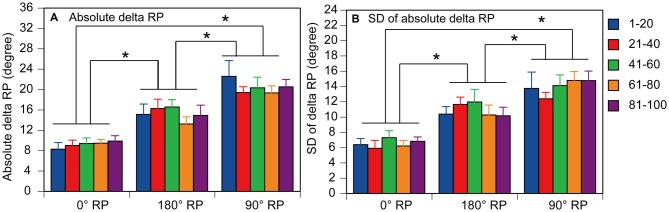
**Absolute delta relative phase (RP; A) and standard deviation of absolute delta (SD of delta RP; B) in the non-TMS session.** Each measure is the average of each of 20 consecutive trials in each trial block. Bars indicate means and error bars indicate standard errors. Asterisks indicate significant differences (*p* < 0.05).

A representative finger movement trajectory is shown in Figure 1. The absolute delta RP was 9.2 ± 0.4° under the 0° RP condition, 15.2 ± 0.6° under the 180° RP condition, and 20.4 ± 0.7° under the 90° RP condition (Figure 6A). ANOVA revealed a significant difference in the absolute delta RP among the tasks [*F*_(2,18)_ = 25.90, *p* < 0.01], but failed to reveal a significant difference among the trial blocks [*F*_(4,36)_ = 0.90, *p* = 0.48] without a significant interaction between the main effects [*F*_(8,72)_ = 1.19, *p* = 0.32]. A *post hoc* test revealed that the absolute delta RP was significantly different between any pair of the tasks (*p* < 0.05).

### Stability of Finger movement

The SD of absolute delta RP was 6.5 ± 0.3° under the 0° RP condition, 10.9 ± 0.5° under the 180 ° RP condition, and 14.0 ± 0.6° under the 90° RP condition (Figure 6B). ANOVA revealed a significant difference in the SD of absolute delta RP among the tasks [*F*_(2,18)_ = 35.71, *p* < 0.01], but failed to reveal a significant difference among the trial blocks [*F*_(4,36)_ = 0.89, *p* = 0.48] without a significant interaction between the main effects [*F*_(8,72)_ = 0.75, *p* = 0.65]. A *post hoc* test revealed that the SD of absolute delta RP was significantly different between any pair of the tasks (*p* < 0.05).

### Pre-Stimulus BEMG

**Figure 7:**
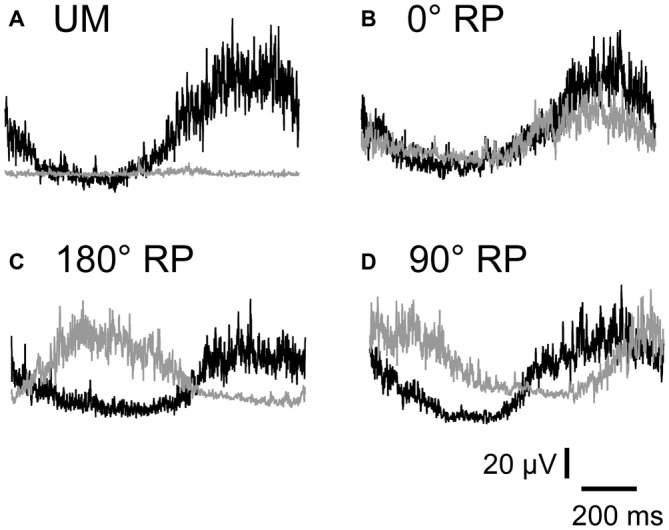
**Specimen record of rectified and averaged electromyo-graphic traces for a single cycle of UM movement (A) and BM movement with 0° (B), 180° (C), and 90° of relative phase (RP; D) in the non-TMS session.** The left end indicates onset of adduction and the right end indicates offset of abduction in the right index finger. Gray traces indicate electromyographic signals in the left first dorsal interosseous muscle and black traces indicate those in the right first dorsal interosseous muscle.

**Figure 8:**
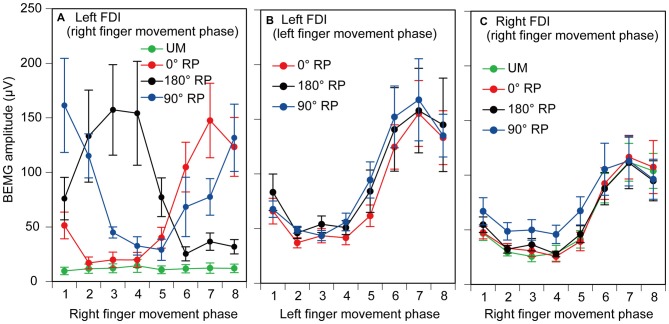
**Pre-stimulus background electromyographic (BEMG) amplitude in the left first dorsal interosseous muscle for each movement phase of the right index finger (A), and that for each movement phase of the left index finger (B), and pre-stimulus BEMG amplitude in the right first dorsal interosseous muscle for each movement phase of the right index finger (C) in the TMS session.** Data points indicate means and error bars indicate standard errors.

EMGs were phase-dependently modulated in the non-TMS session (Figure 7). The pre-stimulus BEMG amplitude in the left FDI muscle for each phase of right index finger movement in the TMS session is shown in Figure 8A. The pre-stimulus EMG amplitude in the left FDI muscle was maximal in the 7th phase of right finger movement and was minimal in the 2nd phase of right finger movement under the 0° RP condition. The pre-stimulus EMG amplitude was maximal in the 3rd phase of right finger movement and was minimal in the 6th phase of right finger movement under the 180° RP condition. The pre-stimulus EMG amplitude was maximal in the 1st phase of right finger movement and was minimal in the 5th phase of right finger movement under the 90° RP condition.

The pre-stimulus BEMG amplitude for each movement phase of the tested finger is shown in Figures 8B,C. The pre-stimulus BEMG amplitude was minimal during the middle of the adduction phase (from the 2nd to 4th phases), and was maximal during the middle of the abduction phase (the 7th phase). ANOVA did not reveal a significant difference in pre-stimulus BEMG amplitude in the left FDI muscle among the tasks [*F*_(2,18)_ = 0.94, *p* = 0.41], but revealed a significant difference among the phases [*F*_(7,63)_ = 10.58, *p* < 0.01] without a significant interaction between the main effects [*F*_(14,126)_ = 0.83, *p* = 0.64]. ANOVA revealed a significant difference in pre-stimulus BEMG amplitude in the right FDI muscle among the phases [*F*_(7,63)_ = 15.38, *p* < 0.01], but did not reveal such a difference among the tasks [*F*_(3,27)_ = 2.55, *p* = 0.08] without a significant interaction between the main effects [*F*_(21,189)_ = 1.31, *p* = 0.17].

### MEP

**Figure 9:**
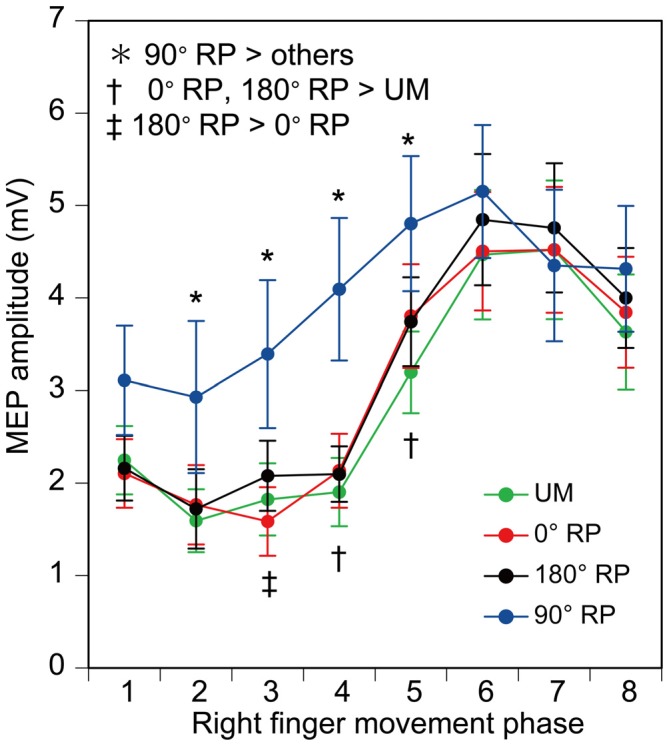
**Amplitude of the motor evoked potential (MEP) in the right first dorsal interosseous muscle.** Bars indicate means and error bars indicate standard errors. Asterisks, daggers, and a double dagger indicate significant differences (*p* < 0.05).

MEP amplitude was minimal in the middle of the adduction phase of right index finger movement (the 2nd phase), and was maximal in the middle of the abduction phase of right index finger movement (the 6th phase), as shown in Figure 9. More importantly, the MEP amplitude during BM movement with 90° RP appeared to be larger than that during the other tasks from the 2nd to 5th phases of right index finger movement. ANOVA revealed a significant difference in MEP amplitude among the tasks [*F*_(3,27)_ = 5.10, *p* < 0.01] and among the phases [*F*_(7,63)_ = 27.10, *p* < 0.01] with a significant interaction between the main effects [*F*_(21,189)_ = 3.23, *p* < 0.01]. A test of the simple main effect revealed a significant difference in MEP amplitude among the tasks in the 2nd [*F*_(3,83)_ = 3.23, *p* < 0.05], 3rd [*F*_(3,83)_ = 7.21, *p* < 0.01], 4th [*F*_(3,83)_ = 13.16, *p* < 0.01], and 5th phases [*F*_(3,83)_ = 6.67, *p* < 0.01]. A *post hoc* test revealed that the MEP amplitude under the 90° RP condition was significantly greater than the amplitude under any of the other movement conditions across the four phases (*p* < 0.05), the amplitude under the 180° RP condition was significantly greater than that under the 0° RP condition in the 3rd phase, and the amplitude under any of the BM conditions was significantly greater than that under the UM condition in the 4th and 5th phases (*p* < 0.05).

**Figure 10:**
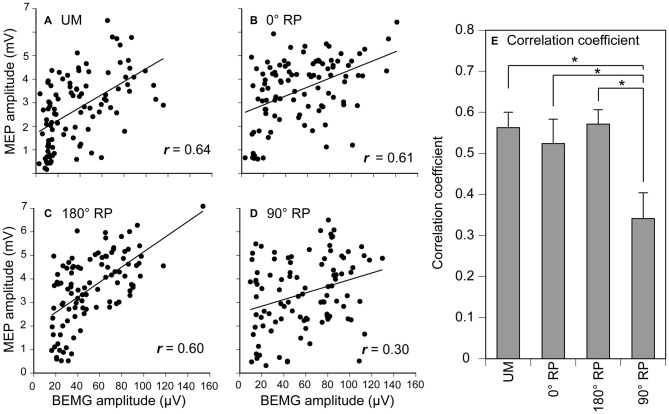
**Scatter plots of the amplitude of the MEP for the amplitude of pre-stimulus BEMG in the right first dorsal interosseous muscle during UM movement (A) and BM movement with 0° (B), 180° (C), and 90° of relative phase (RP; D) in one participant, and the correlation coefficient between MEP and pre-stimulus BEMG amplitudes across the participants (E).** Bars indicate means and error bars indicate standard errors **(E)**. Asterisks indicate significant differences (*p* < 0.05).

**Figure 11:**
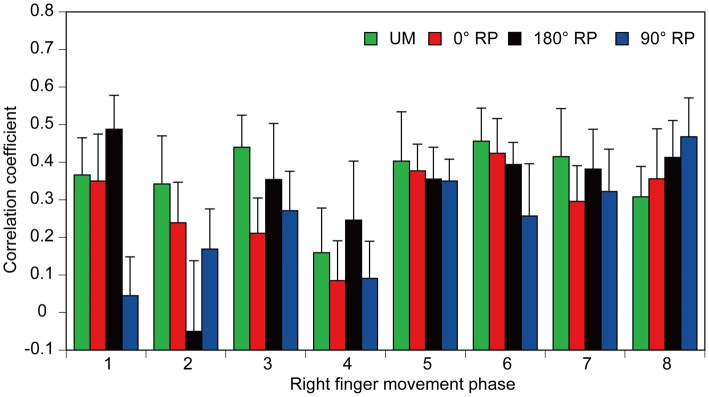
**The correlation coefficients between amplitude of the pre-stimulus BEMG and that of MEP each movement phase of the right finger.** Bars indicate means and error bars indicate standard errors.

A significant positive correlation was found between the pre-stimulus BEMG and MEP amplitudes in the right FDI muscle in each task for each participant (*p* < 0.05), except BM movement with 90° RP in three participants and BM movement with 0° RP in one participant (Figures 10A–D). ANOVA revealed a significant difference in the average correlation coefficient across the participants among the tasks [*F*_(3,27)_ = 6.45, *p* < 0.01] (Figure 10E). A *post hoc* test revealed that the correlation coefficient under the 90° RP condition was significantly smaller than that under any of the other movement conditions (*p* < 0.05). In addition, the correlation coefficient was estimated for each movement phase in each task for each participant (Figure 11). The correlation coefficient was not significantly different among the tasks [*F*_(3,27)_ = 1.07, *p* = 0.38] but was significantly different among the movement phases [*F*_(7,63)_ = 2.55, *p* = 0.02] without a significant interaction between the main effects [*F*_(21,189)_ = 1.32, *p* = 0.17].

## Discussion

In the present study, corticospinal excitability of the hand muscle was observed during UM movement and BM movement with different RPs. The observed RP deviated from the required RP and was unstable during BM movement with 90° RP. Corticospinal excitability was increased and the dependency of corticospinal excitability on the BEMG level was decreased during BM movement with 90° RP.

### Finger Movement

The angle, velocity, and amplitude of the right finger movement were significantly different among the tasks, although those of the left finger movement were not. In the present study, the angle and velocity of finger movement were measured in the TMS session, in which TMS was delivered over the left M1, and thus, motion artifact of the right index finger must have been induced. In a previous study, TMS-induced disruption was different among UM, anti-phase BM, and in-phase BM movements (Chen et al., 2005). Accordingly, one may speculate that the task-dependent right finger movement was due to the above-threshold TMS over the left M1 task-dependently interfering with the ongoing right finger movement. However, this assumption is not likely, because such laterality was present not only for the angle and velocity of finger movement in the TMS session but also for finger movement amplitude in the non-TMS session in which TMS was not given. The participant took some time between the right finger movement and auditory tones during tasks in the present study. Accordingly, an alternative explanation for the task-dependency of right finger movement is that it was due to task-dependent interference from the process of taking time between the finger movement and auditory tones. It is possible that handedness could explain the current results, since the hemispheric asymmetry of cortical activity during hand movement is different between the dominant and non-dominant hands (Kawashima et al., 1993; Alahmadi et al., 2015). In the present study, the participants were right-handed. Thus, the asymmetrical finger movement during BM movement may reflect the hemispheric asymmetry of cortical activity during finger movement.

### Accuracy and Stability of Finger Movements

The absolute delta RP during BM movement with 0° and 180° RP in healthy participants observed in our present study was similar to that in healthy elderly humans, but was smaller than that in patients with Parkinson’s disease (Almeida and Brown, 2013). Moreover, the absolute delta RP and SD of absolute delta RP during BM movement with 90° RP were larger than those during BM movement with the other RPs, which was consistent with previous findings (Yamanishi et al., 1980; Tuller and Kelso, 1989; Zanone and Kelso, 1992; Lee et al., 1995; James et al., 2010). It might be tempting to speculate that acquisition of the ongoing task occurs throughout the experimental session. However, we consider this unlikely, because the tasks were practiced before the experimental session until they were completely acquired, and the SD of the absolute delta RP and the absolute delta RP were not changed across the trial blocks in the session. BM movement with 0° or 180° RP is in the attractor state (Haken et al., 1985), but that with 90° RP is not. Thus, the present findings support the view that BM movement with an RP which is not in the attractor state is less accurate and more variable compared with BM movement with the RP in the attractor state (Yamanishi et al., 1980; Zanone and Kelso, 1992; Lee et al., 1995).

### Task Dependency of Corticospinal Excitability

The MEP during BM movement with 180° RP was slightly larger than that during BM movement with 0° RP in the 3rd phase of right finger movement. This was consistent with a previous study in which MEP in the FCR muscle during bilateral movement with 180° RP tended to be larger than that during bilateral movement with 0° RP (Carson et al., 1999). In a previous study, MEP in the forearm muscle at rest was facilitated by the activity of the contralateral homologous muscle during rhythmic wrist movement (Carson et al., 2004). In the 3rd phase in the present study, during which MEP during BM movement with 180° RP was greater than that during BM movement with 0° RP, BEMG activity of the left FDI muscle was prominent during BM movement with 180° RP but was inactive during BM movement with 0° RP, although that of the right FDI muscle was similar between these movements in this phase. Accordingly, the finding that the MEP in the 3rd phase of right finger movement during BM movement with 180° RP was greater than that during BM movement with 0° RP was likely to be related to the activity of the contralateral homologous muscle.

More apparently, MEP during BM movement with 90° RP was larger than that during the other tasks, indicating that corticospinal excitability increases during BM movement with the RP which is not in the attractor state. This increase was significant from the 2nd to 5th phases, but EMG activity of the tested muscle was inactive in these phases. This means that the corticospinal pathway is restless when the tested muscle is inactive during BM movement with the RP which is not in the attractor state. The MEP amplitude depends on the BEMG level of the tested muscle (Devanne et al., 1997; Hasegawa et al., 2001). However, the BEMG level in the tested muscle is not a major cause of the increase in MEP amplitude during BM movement with 90° RP, because the pre-stimulus BEMG amplitude in the tested muscle during BM movement with 90° RP was not significantly different from that during the other tasks.

There are several possible explanations for the increase in MEP amplitude during BM movement with 90° RP. One is that the increase in the MEP amplitude was due to enhancement of the descending drive to the contralateral homologous muscles. Tonic contraction of the hand muscle facilitated corticospinal excitability in the contralateral homologous muscle (Hess et al., 1986, 1987). In addition, corticospinal excitability in the FCR muscle increased during rhythmic movement of the contralateral wrist, and the increase was prominent in the phase in which the contralateral homologous muscle was active (Carson et al., 2004). Based on this finding, the authors of this previous study proposed that the descending drive to the contralateral homologous muscle is the cause of the increase in corticospinal excitability in the forearm muscle during rhythmic movement of the wrist (Carson et al., 2004). However, this is not likely to be the explanation for our present finding. In the present study, MEP in the hand muscle was facilitated from the 2nd to 5th phases of the right index finger movement, but the pre-stimulus BEMG amplitude in the contralateral homologous muscle during BM movement with 90° RP was larger than that during the other tasks only in the 1st and 8th phases of the right finger movement. Thus, in the phase in which MEP was facilitated during BM movement with 90° RP, the activity of the BEMG in the contralateral homologous muscle was not always prominent. Accordingly, the higher corticospinal excitability during BM movement with 90° RP is not explained by BEMG activity of the contralateral homologous muscle.

The second possible explanation is that the increase in the MEP amplitude is due to lengthening of the tested muscle. The right index finger tended to be adducted during BM movement with 90° RP as compared with that during the other tasks. Adduction of the index finger lengthens the FDI muscle. However, we consider that lengthening of the FDI muscle due to adduction of the index finger must not be the cause of the increase in MEP during BM movement with 90° RP, because lengthening of the muscle actually decreases rather than increases corticospinal excitability (Renner et al., 2006).

The third possible explanation for the increase in the MEP amplitude is task difficulty. BM movement with 90° RP is more difficult than BM movement with 0° or 180° RP (Swinnen and Wenderoth, 2004). This view is supported by the present finding that the number of error trials during BM movement with 90° RP was greater than that during the other movements. When one executes a difficult movement, the amplitude of movement decreases so as to decrease the difficulty of the task by reducing the degree of freedom (Vereijken et al., 1992). The decrease in amplitude of the finger movement during BM movement with 90° RP observed in the present study might also reflect a reduction in the degree of freedom made in order to decrease the difficulty.

Corticospinal excitability increases with increase in task difficulty. In a previous study, corticospinal excitability in the FDI muscle was examined during a precision task with a bandwidth visual feedback of force level (Pearce and Kidgell, 2009). In this previous study, the bandwidth of the boundaries between the correct and incorrect force levels was conditioned; vigorous adjustment of force must have been required when the participants exerted force with a narrow bandwidth of visual feedback. Indeed, corticospinal excitability during force production with a narrow bandwidth of visual feedback was higher than that with a wide bandwidth of feedback, indicating that corticospinal excitability is higher when the participants make a major effort to control the force. From this standpoint, one might speculate that the higher corticospinal excitability during BM movement with 90° RP can be attributed to the greater effort required to control the difficult finger movement. However, other studies have found conflicting evidence against these findings. Asynergistic contractions of the bilateral forearm muscles have been shown to induce the decrease in corticospinal excitability (Leonard et al., 2015). Contraction of the asynergistic muscles in the forearms is more difficult than the mirror contraction of the bilateral muscles. In studies using fMRI, the parietal areas were specifically associated with task difficulty, but the primary motor-sensory areas were not (Wexler et al., 1997; Nair et al., 2003). Based on these previous findings, it cannot be conclusively stated that the higher difficulty of BM movement with the RP which is not in the attractor state is the cause of the higher corticospinal excitability during BM movement with 90° RP.

The fourth possible explanation involves motor binding, which is defined as the central process integrating two or more motor processes into one gestalt, and refers to how movement parts become spatiotemporally united to give rise to the unified experience of coordination (Swinnen and Wenderoth, 2004). Motor binding occurs during BM movement with RP which is not in the attractor state (Swinnen and Wenderoth, 2004). Indeed, BM movement with 90° RP is easier to execute when performed in accordance with visual feedback of the lissajous figure of actual finger movements (Kovacs and Shea, 2011). This is reasonably explained by the view that two task components, left and right finger movements, are integrated into a gestalt via visual feedback of the finger movement trajectory. In the present study, the participants executed BM movement through visual feedback of the lissajous figure of actual finger movements, and thus such motor binding must have occurred. The M1 plays a role for motor binding integrating a complex motor plan (Sanes and Truccolo, 2003). Therefore, the higher corticospinal excitability during BM movement with 90° RP might be explained by an increase in excitability of the M1, which reflects the motor binding process.

The fifth possible explanation is involvement of the interhemispheric interaction during BM movement with the RP which is not in the attractor state. Interhemispheric interaction is involved in control of BM movement with the RPs which are not in the attractor state (Tuller and Kelso, 1989). The M1s are interconnected by the corpus callosum mediating interhemispheric interaction (Wahl et al., 2007). Indeed, interhemispheric inhibition mediated by the corpus callosum is enhanced during BM coordination of force (Hiraoka et al., 2014). Accordingly, the higher corticospinal excitability during BM movement with the RP which is not in the attractor state may reflect involvement of the interhemispheric interaction.

The most likely explanation for the increase in corticospinal excitability is that the activity of the M1 increased due to the practice of BM movement with 90° RP. As shown in a previous study, acquisition of BM movement with 90° RP is completed before 80–100 trials of practice (Debaere et al., 2004; Rémy et al., 2008). In contrast, motor learning is not required for BM movement with the RP in the attractor state (Zanone and Kelso, 1992). Some cortical areas are modulated by the practice of BM movement with 90° RP; activities of the dorsolateral prefrontal cortex, premotor cortex, superior parietal cortex, and cerebellum are decreased, but those of the basal ganglia, hippocampus, superior temporal gyrus, and cingulate motor cortex are increased after practice of BM movement with 90° RP (Debaere et al., 2004; Rémy et al., 2008). More importantly, the activity of the primary motor cortex increased after practice of BM movement with 90° RP, but no such increase was observed after practice of BM movement with 0° RP (Debaere et al., 2004). In the present study, practice was continued until the tasks were completely performed. Acquisition of the task was certain, because the absolute delta RP and SD of the absolute delta RP were not significantly changed across the trial blocks in the experimental session. Based on these facts, it is plausible that acquisition of the task through practice caused an increase in corticospinal excitability specifically during BM movement with 90° RP.

### Dependency of MEP on BEMG Level

In the present study, the correlation coefficient between MEP and pre-stimulus BEMG amplitudes across the movement phases during BM movement with 90° RP was significantly smaller than that during the other tasks. Accordingly, modulation of MEP associated with the changes in EMG activity level across the movement phases was specifically weak during BM movement with the RP which is not in the attractor state. Despite this finding, the correlation coefficient between these parameters for each movement phase was not significantly different among the tasks. The different findings for the correlation coefficient across the movement phases and that for each movement phase were attributed to the fact that a substantial modulation of the EMG activity level does not occur within each movement phase but occurs across the movement phases. The muscle activity level is under control of the descending drive to the muscle through the corticospinal pathway, because MEP is highly dependent on EMG activity (Hasegawa et al., 2001). Thus, the present findings support the view that the contribution of the descending drive to the hand muscle through the corticospinal pathway is minor during BM movement with the RP which is not in the attractor state.

### Limitations

One limitation of the present study is that TMS intensity was not determined by target MEP amplitude but by RMT. For this reason, the MEP amplitude tested was not consistent across the participants in the present study. Susceptibility of the MEP is dependent on its size. Thus, inter-participant variability of the MEP amplitude may have affected the susceptibility of the MEP. Moreover, the MEP amplitude was not normalized in the present study. In previous studies, the MEP amplitude has been normalized by maximum M-wave amplitude, MEP amplitude at rest, or a mathematical technique for excluding inter-participant variability of MEP size (Perez et al., 2004; Maioli et al., 2007; van Elswijk et al., 2008). In the present study, the raw MEP amplitude was used for statistical analysis. This analytical procedure applied in the present study may also have affected the susceptibility of the MEP.

Hemispheric asymmetry of cortical activity is present during hand movement (Kawashima et al., 1993; Alahmadi et al., 2015). In the present study, finger movement was asymmetrical during BM movement, and the participants were right-handed. Thus, handedness is a possible cause of the findings during BM movement. In spite of this, statistical analysis was conducted for each finger independently, because of the asymmetrical (data was analyzed only under the BM conditions in the left finger but under both the UM and BM conditions in the right finger) or incompatible data sets (the BEMG data in the left and right FDI muscles were recorded from different pairs of electrodes). Thus, the present study did not elucidate the effect of handedness on the motor process of BM movement. Further investigations are needed on this issue.

## Conclusion

Corticospinal excitability was increased during BM movement with 90° RP. This increase may have been caused by the excessive effort required to control BM movement with the RP which is not in the attractor state, the involvement of interhemispheric interaction, the motor binding process, or acquisition of the task. The dependency of corticospinal excitability on the BEMG activity level was decreased during BM movement with 90° RP, indicating a minor corticospinal contribution to BM movement with the RP which is not in the attractor state.

## Author Contributions

YN and KH experimental design, conduct experiment, analysis, writing article. YJ, KT, and YC conduct experiment, analysis.

## Conflict of Interest Statement

The authors declare that the research was conducted in the absence of any commercial or financial relationships that could be construed as a potential conflict of interest.
